# Factors influencing adolescent girls’ decision in initiation for human papillomavirus vaccination: a cross-sectional study in Hong Kong

**DOI:** 10.1186/1471-2458-14-925

**Published:** 2014-09-08

**Authors:** Albert Lee, Mandy Ho, Calvin Ka Man Cheung, Vera Mei Wen Keung

**Affiliations:** Centre for Health Education and Health Promotion, The Chinese University of Hong Kong, 4th Floor Lek Yuen Health Centre, Shatin, New Territory, Hong Kong; Department of Applied Health Science, Bloomington School of Public Health, Indiana University, Bloomington, USA; The Children’s Hospital at Westmead Clinical School, University of Sydney, Locked Bag 4001, Westmead, NSW 2145 Australia

**Keywords:** Cervical cancer, Cancer prevention, Human papillomavirus (HPV) vaccine, Chinese girls, School health education, Community health education

## Abstract

**Background:**

Cervical cancer is one of the common cancers among women worldwide. Despite HPV vaccination being one of the effective preventive measures, it is not included in government vaccination programme in Hong Kong. This study aimed to assess the knowledge of and attitude towards cervical cancer prevention among Chinese adolescent girls in Hong Kong, and to identify factors influencing the initiation of HPV vaccination.

**Methods:**

This was a cross-sectional study conducted in Hong Kong during the period of October 2010 to November 2010. A self-administered questionnaire was used, with 1,416 girls from 8 secondary schools completing the questionnaire. Knowledge scores were composited and initiation of HPV vaccination was staged based on stage of change. Analyses were conducted to identify the association of initiation of HPV vaccination with participant’s personal and family factors as well as their knowledge and attitude towards cervical cancer prevention.

**Results:**

The uptake rate of HPV vaccination was low (7%) with 58% respondents in pre-contemplation and contemplation stage. The survey identified a significant gap in knowledge on cervical cancer prevention. The main channels of information were from media and very few from schools or parents. However, 70% expressed their wishes to have more information on cancer prevention, and 78% stated that they were willing to change their lifestyles if they knew the ways of prevention. Multivariate analysis identified three independent significant factors for initiation of vaccination (action and intention): perceived cancer as terrifying disease, school should provide more information on cancer prevention, and comments from relatives and friends having received the vaccine. The cost of vaccination and socio-economic background were not found to be significant.

**Conclusions:**

Public education on cervical cancer needs to be well penetrated into the community for more sharing among friends and relatives. School as setting to provide source of information would facilitate uptake rate of HPV vaccine as students have expressed their wishes that school should provide more information on prevention of cancer. School and community education on cancer prevention would help adolescents to have better understanding of the seriousness of cancer.

**Electronic supplementary material:**

The online version of this article (doi:10.1186/1471-2458-14-925) contains supplementary material, which is available to authorized users.

## Background

Cervical cancer is the fourth commonest cancer among women worldwide with estimated 528,000 new cases in 2012 [1], and the eleventh commonest cause of cancer-related death among women in Hong Kong (HK) in 2011 [2]. Virtually all cervical cancers are caused by persistent infection with human papillomavirus (HPV), particularly HPV types 16 and 18. HPV vaccine is highly effective in preventing persistent HPV infection and precancerous cervical cancer caused by HPV-16 and HPV-18 among females not yet infected at the time of vaccination [3, 4]. The cumulative lifetime risk of infection can be greater than 75% for one or more genital HPV infection [5]. In Hong Kong, about 10% of young women (under 26 years old) were HPV positive [6]. Recent study in Macao (neighbouring city of Hong Kong) showed higher prevalence of HPV infection and the most prevalent genotypes were found to be similar to those in south-west and southern China [7]. There has been increasing rates of cervical cancer among younger generation in China and also leading causes of premature death among women in south Asia, [8], prevention is an important public health issue so study on the drivers and barriers to HPV vaccination is very much needed [9].

Studies conducted during the early phase of introduction of vaccine including studies conducted in HK among Chinese girls attending secondary school (age 12–19), revealed that intention to be vaccinated depended on understanding of HPV being the causative agent, safety and efficacy of vaccination, perceived seriousness of HPV infection, the cost of the vaccine, and recommendation by general practitioners (GPs) or significant others such as family members [10–13]. Systematic review conducted in Australia covering studies from 2006 to 2011 highlighted the importance of correct information by a reliable source such as health care providers and higher vaccine uptake rate by school based vaccination [14]. The authors called for more studies from a boarder range of developed and developing countries. However, very few studies had targeted Chinese population. Although only 15% of women living in metropolitan and rural regions of China had heard of HPV vaccine, they were willing to be vaccinated if available [15]. Study among university students in HK (mean age 19.4 ± SD 1.0 years) has shown that sexually active participants of the study reported greater interest in HPV vaccination [16]. Sexual openness amongst Chinese adolescents has increased during recent years [17]. The first peak of cervical cancer has been shown at age 40 which suggested that females in Hong Kong would be infected by HPV during early 20s [18]. As immune response to HPV vaccination is higher in younger subjects [19], we need to examine the determinant factors for initiation of HPV vaccine of this age group.

Currently in Hong Kong, HPV vaccination is not included in government vaccination programme (quadrivalent vaccine was first licensed in Hong Kong) so recipients are required to pay about HK$3,000 (US$1 = HK$7.8) to complete the 3 doses from GPs privately. The GDP per capita in Hong Kong is comparable to USA (US$36,667 and US$49,922, respectively, according to International Monetary Union, 2012). The Centre for Health Protection of Department of Health has issued guidelines for cervical cancer prevention covering cervical cancer screening and HPV vaccine, However many factors such as cost, understanding and attitude towards cervical cancer can affect adolescent girls’ decision of receiving HPV vaccination. Study in 2010 revealed that only 11% of girls had intention to have vaccination. This study aimed to update the potential factors influencing the initiation of HPV vaccination among Chinese adolescent girls in HK without universal coverage. The findings would enlighten future strategy to improve the vaccine uptake rate with cost being a barrier. Although the uptake of HPV vaccine might be influenced by socio-cultural context, the results of this study will be useful not only for Asian countries and also other countries without full coverage of vaccination.

## Methods

### Study design and sampling method

This was a cross-sectional study conducted in Hong Kong during the period of October 2010 to November 2010. The participants were female secondary school students studying secondary two, S2 to secondary seven, S7 in age range 12 to 19. Invitation was sent to eleven secondary schools which had once participated in recent programs organized by Centre for Health Education and Health Promotion of The Chinese University of Hong Kong (CUHK). Eight schools located in different regions of HK consented to participate in the survey. One class of students from S2 to S5 was randomly selected in each school to participate while all female students from S6 to S7 were recruited to participate due to smaller class size (the class size of S6 and S7 is smaller and just over half of S5 students would be promoted to S6).

### Ethics statement

Written consent from parents and assent from adolescent girls were obtained prior to filling out the anonymous questionnaire. Ethical approval was granted by the Survey and Behavioural Research Ethics Committee of the Chinese University of Hong Kong.

### Data collection and measuring instrument

The self-administrated questionnaire used in the study was developed with reference from previous relevant studies in Hong Kong [12, 13, 16] with inputs from experts in adolescent preventive medicine for content validation. It was then pilot tested for face validity on a convenient sample of 15 female students studying in S4 and S5. Questionnaires together with an instruction guide detailing the study procedures and points to note were sent to the teacher in charge of the participating schools. Under the arrangement of a school teacher, students were required to fill out the questionnaire in class and on their own, and no guidance or discussion of answers was allowed during the study. Upon receiving the completion notice from schools, researchers visited the participating schools to collect all the questionnaires sent, including both the completed and blank questionnaires. The questionnaire aimed to collect data reflecting the factors having impact on initiation of HPV vaccine such as students’ awareness and knowledge of cervical cancer, HPV infection and HPV vaccine; their attitudes and beliefs towards cervical cancer prevention; and other socio-demographic factors influencing the initiation of vaccination for adolescents. There were altogether 22 items and questionnaire design was based on theoretical framework of Health Belief Model focusing on perception of severity, susceptibility, barriers and benefits [20], the Theory of Reasoned Action focusing on attitudes and social norm [21], and social cognitive theory focusing on external determinants of behaviors apart from attitudes, values and belief [22]. There were three sections and the contents were described as follows:

#### Section 1 cancer and cancer prevention (9 items)

Section 1 started with 3 multiple choice questions evaluating participants’ general knowledge on cancer prevalence and mortality in HK. It was followed by 5 questions exploring participants’ attitude towards cancers and cancers prevention using a 6-point likert scale (1 = strongly disagree, 6 = strongly agree) and an assumptive question exploring the different possible considerations for uptake of a preventive vaccination using 4-likert scales from ‘very unimportant’ to ‘very important’.

#### Section 2 cervical cancer (8 items)

Section 2 started with a question on participants’ awareness of cervical cancer. Participants who had heard of cervical cancer, were directed to answer the remaining questions in this part, which included 6 questions adopted from HK Family Planning Association [12] testing their general understanding of cervical cancer. This short knowledge test had been reviewed based on the inputs from experts in adolescent preventive medicine, and the pilot testing on a sample of female students studying in S4 and S5 had further validated the questions. Choices including ‘Yes/No/Don’t know’ were provided, with each correct answer receiving 1 score while incorrect answer or ‘don’t know’ receiving 0 score with equal weighting for each question like previous study [12]. The knowledge test began with 2 basic questions on cervical cancer, followed by 4 more challenging questions to enhance the discriminative power of the test. A knowledge score (ranged from 1 to 6) was calculated by adding up the scores of the 6 questions (Additional file 1). Answers to each of the 6 questions were found to be moderately to strongly correlated (r = 0.337-0.604) with the total score of participants in our study, and significant difference was also found for all questions between those who scored higher and lower in total knowledge score (p < 0.01), indicating the ability of the 6 questions to effectively discriminate the knowledge on cervical cancer between the stronger and the weaker ones among secondary students. The last question asked participants about channels through which they learnt about cervical cancer.

#### Section 3 HPV vaccine (5 items)

Section 3 started with a question on participants’ awareness of HPV vaccine. Participants heard of the vaccine, were directed to answer the remaining questions which included channels through which participants learnt about HPV vaccine, knowledge and intention towards vaccination adopted from a previous study on University students with slight modification to suit the secondary students’ level [16]. The questions about intention towards vaccination provided 4 response choices (have received HPV vaccine; considering to be vaccinated; required more information to decide; will not consider/ has never considered to be vaccinated) to reflect the stage of change broadly categorized as pre-contemplation and contemplation stages (still passive), and intention and action stages (more pro-active) (Additional file 1) [23]. Participants also completed a question regarding the drivers and barriers to HPV vaccination.

Demographic information including the age of participants, parents’ education level, marital and working status, and the monthly family income were also collected.

### Data analysis

Descriptive data from all variables in the questionnaire were computed. Each variable explored using Likert scale characterized the respondents’ attitude and was treated as attitude score during analyses. Knowledge scores were composited from the six questions reflecting participants’ understanding of cervical cancer and questions indicating their consideration of vaccination reflected the stage of change (Additional file 1). All demographic variables of respondents were used to investigate their association with students’ knowledge and attitude towards cancers, and initiation of HPV vaccination. Those variables not on Likert scale were treated as categorical variables. Chi-square test was used to analyze factors associated with initiation of HPV vaccination. T-test was applied to compare the knowledge and attitude score towards cancer and cancer prevention between those who were in pre-contemplation/contemplation stage and those who were in intention/action stage for HPV vaccination. As HPV vaccine is still not universally available, behavioural intention is an important outcome measurement as well as actual uptake rate. Logistic regression was conducted with action and intention as dependent variable reflecting proactive move while contemplation and pre-contemplation as reference category reflecting the passive motion to identify which independent variables predicted the initiation of HPV vaccination. The independent variables included demographic, personal and family factors, perception of vaccine and the scores of knowledge and attitudes. The scores of knowledge and attitudes were re-categorised as binary variables with answering half or more questions correctly as one category and less than half as reference. The multi-variate analysis was conducted with stepwise approach. All data analyses were performed using SPSS version 14.0. A p value of less than 0.05 was considered to be statistically significant.

## Results

Total of 1,416 questionnaires were returned from eight participating schools with a response rate of 84.4%. Among the questionnaires received, 2 were blank questionnaires so the total valid response was 1,414. The age of the respondents ranged from 13 to 21 years old (mean = 16.53, SD = 1.95). Table 1 summarizes the demographic characteristics of the subjects with reference to Hong Kong population in 2010.

**Table 1:** **Demographic characteristics of respondents in comparison to Hong Kong population 2010**

Characteristic	Number of respondents (%)	Hong Kong population 2010 ^#^
**Age** (n = 1385)		Enrolment of female in secondary day schools by age
13-15	454 (32.8)	108,749 (58.7%)
16-18	705 (50.9)	69,976 (37.8%)
19-21	226 (16.3)	6,589 (3.6%)
**Parental education -** Father (n = 1175)		Distribution of population aged 15 and over by educational attainment
Primary	243 (20.7)	540,253 (18.8%)
Secondary	749 (63.7)	1,533,821 (53.4%)
Post-Secondary	183 (15.6)	801,064 (27.9%)
**Parental education -** Mother (n = 1210)		
Primary or below	245 (20.2)	850,743 (25.4%)
Secondary	840 (69.4)	1,713,905 (51.2%)
Post-Secondary	125 (10.3)	782,435 (23.4%)
**Parental marital status** (n = 1384)		
Married/cohabited	1218 (88)	
Divorced/separated	121 (8.7)	
Widowed	45 (3.3)	
**Working status of father** (n = 1243)		Labour Force (thousands) and Labour Force Participation Rates
Full time	1080 (86.9)	Male: 1 940.9 (68.6%)
Part time	44 (3.5)	
Housekeeping/unemployed/Retired	119 (9.6)	
**Working status of Mother** (n = 1359)		
Full time	643 (47.3)	Female: 1 712.8 (52.0%)
Part time	149 (11)	
Housekeeping/unemployed/Retired	567 (41.7)	
**Monthly family income** ^1^ (n = 786)		Domestic Households by Monthly Household Income (thousands)
HK$9999 or below	167 (21.2)	629.2 (27.0%)
HK$10 000 - $14 999	173 (22)	346.3 (14.8%)
HK$15 000 - $29 999	326 (41.5)	692.0 (29.7%)
HK$30 000 or above	120 (15.3)	666.6 (28.6%)

^1^US$1 = HK$7.8.

^**#**^Census and Statistics Department, Hong Kong Special Administrative Region (2011). http://www.statistics.gov.hk/pub/B10100032011AN11B0100.pdf.

### Knowledge of cervical cancer and preventive measures

Majority of the participants (95.9%) had heard of cervical cancer. Most respondents were aware that cervical cancer was a common cancer among women (92.9%), yet around half did not seem to have thorough understanding of its risk factors, including that HPV is a causative factor (41.1%), having multiple sex partners increases the likelihood of cervical cancer (48.2%), and cervical cancer can develop in any women with sexual activity (52.5%). Only half knew about Pap smear screening as a preventive measure (52.8%). About one third had misconception of prognosis with 37.3% of respondents stated that cervical cancer could hardly be treated. Regarding HPV vaccination to prevent cervical cancer, 71.9% of respondents were aware of it. The commonest channels of information were television (TV) (28.3%) followed by outdoor advertisement (12.5%), clinic/hospital (11.1%), newspaper (9.7%), schools (9.5%), magazine (7.1%) and internet (6.8%). Only 5.7% reported learning about it from parents or siblings.

### Attitudes towards cancer prevention

Majority of respondents (87.4%) had the feeling that cancer is a terrible disease. Nearly 70% expressed their wishes to have more information on cervical cancer prevention, 71% respondents thought schools should provide more information to enhance their knowledge on prevention of cancer, and they were willing to change their lifestyles if they knew the ways of prevention (78.3%). Less than half of respondents (41.5%) felt that Hong Kong has provided adequate services to promote prevention of cancer.

### Factors affecting vaccination uptake

**Figure 1:**
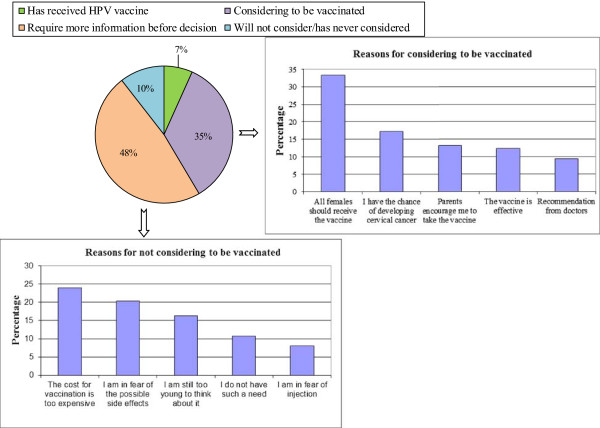
**Initiation of HPV Vaccine (n = 1038), reasons for and against intention to be vaccinated.** Only small proportion of respondents (6.7%) had received HPV vaccination, with nearly half of the respondents (48%) wanting to know more before deciding. Among the ones who refused to consider vaccination, the cost, potential side effects, age, perceived needs and fear of injection appeared to be their main concerns. On the other hand, the perceived risk of getting cervical cancer, recommendations from credible sources including doctors and parents, and the efficacy of the vaccine were the more important considerations among the ones intending to be vaccinated.

About one third of respondents (33.5%) felt that all females should be vaccinated. Figure 1 shows 6.7% vaccination uptake rate (action), 34.8% intended to be vaccinated, and nearly 60% of respondents remained in contemplation (requesting more information before decision) and pre-contemplation stage. Cost of vaccination, side effects, being too young for vaccination, low perceived needs and fear of injection were raised as the most important concerns for not considering (Figure 1). To those respondents who intended to be vaccinated, perceived risk of developing cervical cancer, recommendation from doctors, support from parents, and effectiveness of the vaccine were reported to be important considering factors.

### Association between knowledge scores and personal and family factors

Students in higher grades were found to have higher score by both uni-variate and multi-variate analysis (p < 0.01) (Table 2). Family income, parents’ education level, and employment status were not associated with students’ knowledge on cervical cancer. Channels of information from newspaper, outdoor, internet and school were found to be associated with higher knowledge score with statistical significance by uni-variate analysis and information from schools and newspaper were found to be statistical significance by multi-variate analysis (Table 2). Significant difference in knowledge score was also found between those regarding cancer as terrifying disease compared with those who did not (p <0.01), and also those wishing to know more about cancer prevention in contrast to those not wanting to know more (p = 0.01).

**Table 2:** **The factors identified to be associated with mean knowledge score**

Factors	Mean knowledge score ^#^		Univariate analysis		Multivariate analysis	
			Mean difference (95% CI)	P Value	Unstandardized coefficients (95% CI)	P value
Grade	S5-S7	S2-S4	0.38 (0.22-0.54)	<0.01*	0.35 (0.16-0.55)	<0.01*
	3.4	3				
Information from	Newspaper	No Newspaper	0.19 (0.03-0.35)	0.02*	0.28 (0.09-0.46)	<0.01*
	3.36	3.16				
Information from	Internet	No Internet	0.17 (0.01-0.34)	0.04*	/	/
	3.36	3.19				
Information from	Outdoor advertisement	No Outdoor advertisement	0.19 (0.03-0.34)	0.02*	/	/
	3.35	3.16				
Information from	School	No School	0.41 (0.24-0.58)	<0.01*	0.49 (0.29-0.69)	<0.01*
	3.53	3.12				
Cancer is a terrifying disease	Agree	Disagree			0.19 (-0.02-0.40)	0.07
	3.28	2.94	0.34 (0.10-0.59)	<0.01*		
I wish to have more information on cancer prevention	Agree	disagree	0.24 (0.05-0.42)	0.01*	/	/
	3.31	3.1				

#No significant difference in mean knowledge score was found for factors including parents’ working status and education level, family income, living with parents, source of information on HPV from TV, radio, magazine, hospital/clinic, relatives and friends, and agreement on the following statements: School should provide more information on cancer to enhance the knowledge of students, Hong Kong has provided adequate services in promoting prevention of cancer and If I know any ways which can prevent cancer, I am willing to change in an attempt to put them into practice.

*= P value with statistical significance.

### Factors affecting intention of vaccination

Table 3 provides an uni-variate analysis of association between personal and family factors, and intention of vaccination. Those in intention and action stages had significantly higher percentages of mothers with education level secondary school or above and family income of HK$30,000 or above (P = 0.03). On the other hand, those considered reasonable cost of vaccine (P = 0.02) and seeking comments from relatives and close friends (P = 0.03) had higher percentage in contemplation and pre-contemplation stage (Table 3). The action and intention group had higher knowledge score than the contemplation and pre-contemplation group with statistical significance (P = 0.04) (Table 4). The students in action and intention stage had significant higher scores in rating cancer as terrible disease (P = 0.04) and more willing to make changes after knowing the ways of preventing cancer (P = 0.02) (Table 4).

**Table 3:** **Association of demographic, personal/family factors and perception of vaccine with initiation of HPV vaccination**

	Intention/Action (%)	Contemplation/Pre-contemplation (%)	P value
Mother education level secondary or above	313 (N = 374) (83.7%)	414 (N = 531) (77%)	0.03*
Family income > HK$30,000	107 (N = 253) (42.3%)	168 (N = 532) (31.6%)	0.03*
Reasonable cost of vaccine	361 (N = 427) (84.5%)	542 (N = 606) (89.4%)	0.02*
Comments from relatives and close friends	299 (N = 427) (70%)	470 (N = 606) (78%)	0.01*

N = Number of respondents in the Intention/Action group or Contemplation/Pre-contemplation group with valid data.

% = Percentage of respondents who met the study factor within the group.

*= P value with statistical significance.

**Table 4:** **Association of knowledge and attitude related factors with initiation of HPV vaccination (N = Total numbers of subjects with valid data under each category of answer)**

	Intention/Action stage ^#^	Pre-contemplation/Contemplation stage ^#^	Mean difference [95% CI]	P value
Mean knowledge score (N = 1343)	3.52	3.33	0.18 [0.01-0.37]	P = 0.04*
I think that Cancer is a terrifying disease (N = 1409)	5.11	4.97	0.147 [0.01-0.285]	P = 0.04*
If I know any ways which can prevent cancer, I am willing to change in an attempt to put them into practice (N = 1409)	4.56	4.4	0.17 [0.03-0.3]	P = 0.02*

#No statistical significance in mean knowledge score between the two stratified groups was found for agreement to the following statements: I think schools should provide more information on cancer to enhance the knowledge of students, I think that Hong Kong has provided adequate services in promoting prevention of cancer and I wish to have more information on cancer prevention.

*= P value with statistical significance.

Logistic regression showed that perceiving cancer as a terrifying disease, seeking comments from relatives and close friends, and school should provide information on cancer prevention were found to have significant correlation with initiation of HPV vaccination (Table 5).

**Table 5:** **Multi-variate analysis on factors associated with initiation of HPV vaccination (N = Total numbers of returned questionnaires with valid data of that particular variable)**

Factors	Odds ratios (95% CI)	P value
**Mother education (N = 905)**		
Primary or below	1	
Secondary	0.61 (0.2, 1.87)	0.38
Post-secondary	1.66 (0.61, 4.55)	0.32
**Income (N = 785)**		
HK$9999 or below	1	
HK$10 000 - $14 999	0.68 (0.26, 1.79)	0.43
HK$15 000 - $29 999	0.87 (0.37, 2.01)	0.74
HK$30 000 or above	0.87 (0.36, 2.1)	0.76
**Reasonable cost for vaccine (N = 1033)**		
Not important	1	
Important	0.676 (0.29, 1.60)	0.37
**Comments from relatives and close friend**		
**(N = 1.033)**	1	
Not important Important	1.9 (1.04, 3.49)	0.04*
**School should provide more information for students on cancer prevention (N = 1407)**		
Disagree	1	
Agree	4.35 (1.09, 17.3)	0.04*
**Cancer is a terrifying disease (N = 1409)**		
Agree	1	
Disagree	0.1 (0.016, 0.63)	0.01*
**Understanding of cervical cancer (6 questions, Additional file** **1** **) (N = 1343)**		
Answering correctly 2 questions or less	1	
Answering correctly 3 questions or more	1.11 (0.56, 2.17)	0.77

* = P value with statistical significance.

## Discussion

This study provided updated information on the uptake rate of HPV vaccination in Hong Kong, knowledge and attitude of adolescent girls towards cervical cancer prevention, as well as the drivers and barriers to HPV vaccination. The findings would be useful for the development of future strategies to improve vaccine uptake rate. The uptake rate of HPV vaccination was low with over half of the respondents in pre-contemplation and contemplation stage, and there was a knowledge gap on cervical cancer prevention. Knowledge scores, reasonable cost of vaccination, parents’ education level, family income, comments from friends and relatives, perception of cancer being horrifying disease, and knowing more about the linkage of HPV infection to cancer prevention were found to be significant factors for initiation of vaccine by uni-variate analysis. Multivariate analysis identified three independent significant factors associated with initiation of vaccine: perception of cancer as terrifying disease, school should provide more information on cancer prevention and comments from relatives and friends having received the vaccine.

Social norm and social influences and also self-perceived susceptibility were the main determinant factors for initiation of HPV vaccination as reflected by reporting the importance of parental support, recommendation from doctors, having all females to receive the vaccine, and high likelihood of developing cervical cancer, which are similar to findings in previous studies conducted locally and overseas [11–14]. However, adolescent girls reported that the main channels of information were from media and very few received information from schools or parents which could possibly be one of the contributing factors for the low HPV uptake rate. Those in contemplation and pre-contemplation stages perceived fewer benefits and more harms towards HPV vaccination as reflected by their own values and external factors such as influence from significant others and social norms [20–22]. Channel of information from media would become a source of social influence so it would also make a change to the knowledge of cervical cancer prevention. Although study findings revealed an overwhelming desire for more information on cervical cancer prevention by the respondents and their reported willingness to change their lifestyles for prevention, they did not receive much information and offer of HPV vaccination services in clinical setting so the uptake rate remained low (Figure 1). If the adolescent health providers could discuss disease prevention and side effects of vaccination, the uptake rate would be improved as reflected by a recent survey conducted amongst parents and adolescents aged 15 to 17 in a county of New York [24].

Multivariate analysis identified three important independent factors associated with initiation of vaccination (Table 5). As the actual uptake rate of vaccination was low among respondents and also among female population in Asia [25], comments from close friends and relatives became a strong driving force to parents’ decision and also the significant source of information regarding benefits and side effects of vaccination which explained why it was found to be a significant independent factor. A recent qualitative study in HK [26] revealed lack of recommendations from health care providers so comments from friends and close relatives would then become critical. The active involvement of GPs and other health professionals in cervical cancer prevention would help to build up a critical mass of population with a more positive attitude towards HPV vaccine. This would have significant impact on their close friends and relatives by sparking off the cascade effect.

Reinforcement of vaccination through social network was also found to have core influence on parental support for vaccination when challenged by anti-vaccination messages [27]. Mothers used a simple risk and benefit equation when they encountered ‘anti-vaccination’ messages, so advocacy for vaccination should involve stories about people benefited from vaccine-preventable diseases [28]. This would explain why the perception of cancer as terrifying disease turned out to be the independent significant factor. In one UK study, it was found that mothers with experience of cancer in the family were more likely to accept HPV vaccination for their pre-pubertal daughters [29].

To facilitate the initiation of HPV vaccine among students, schools should provide information and this factor is independent significant factor for initiation of HPV vaccination. Many studies have shown the effectiveness of school based health promotion in improving health behaviours of student [14, 30, 31]. Hong Kong has established a good infrastructure of ‘Health Promoting School’ based on WHO standard and has demonstrated the effectiveness of health improvement among student [31]. Schools in Hong Kong do not have school nurses to deliver information on effective cervical cancer prevention. If school staff can be equipped with appropriate knowledge, they are the potential health advocates whom students would turn to for information [32]. In HK, there is room to equip the knowledge of epidemiology of HPV infection and skills in motivating for behaviours change among GPs [33]. Systematic reviews have revealed higher acceptance of HPV vaccine among parents and young adults who believed that their physicians would recommend [14, 34, 35]. The local GPs are the potential source of information on health issues, and would improve the health literacy of both students and family members based on the conceptual model of health literacy at risk [36].

There were some of limitations of the current study that should be acknowledged. Firstly, the participating schools were not recruited based on random sampling but the sample size was larger than other related studies in Hong Kong. Table 1 shows how our sample population compared with Hong Kong population. Our sample consisted of higher proportion of girls aged 19 or above still in secondary school. The sample population had lower proportion of parents with post-secondary school education and income above US$3,750. Our study captured those with family income between US$1,250 to US$3,750 (above poverty line and below median level), and also those students from schools willing to participate in cervical cancer prevention program and the sample was not skewed towards higher socio-economic class. Many studies on this topic used convenient sampling method [17] with very few studies of sample size over 1,000 on Chinese female adolescents. Secondly, a cross sectional study would only identify associating factors rather than causative factors. Future study has been planned to follow up the vaccination behavior. Thirdly, variables reflecting demographic, family and personal factors were found to have higher percentage of missing data. This might have some effect on the statistical power in demonstrating significant difference. There were some missing data in some questions but the numbers were small (less than 20 questionnaires). The demographics (including parents’ working status, parents’ education level and family income) between the participants who answered and did not answer three of the particular questions (ever heard of cervical cancer, ever heard of HPV vaccine and intention for vaccination) were compared with no statistical difference (P above 0.5), indicating low likelihood of responding bias.

The classes of S6 and S7 were smaller which would introduce a selection bias in the older age group. Sub-analysis of differences in knowledge scores and demographic factors between students of S6 to S7 and S2 to S5 was performed with no statistical significance. Sexual activities were not recorded as schools did not want possible misconception of implication of HPV vaccine to early sexual activity.

### Implications for school health

The school should provide more information on prevention of cancer and also enhance the health education so students have correct understanding of the seriousness of cancer.

### Implications for general practitioners/primary healthcare providers

Primary healthcare providers especially general practitioners are credible sources of health information parents and students tend to trust. By providing reliable information regarding cervical cancer prevention and HPV vaccine via different media or even daily practices, primary healthcare providers can play a leading role in helping the public develop positive images about vaccination for cancer prevention, which would likely enhance the uptake rate of HPV vaccine.

## Conclusion

Public education on cervical cancer needs to be well penetrated in the community as comments from relatives and friends play a significant role in influencing initiation of vaccine. If the adolescents do not agree that cancer as terrifying disease, they are unlikely to consider HPV vaccine to prevent cervical cancer. School as setting to provide source of information would be one strategy to facilitate uptake rate of HPV vaccine as students have expressed their wishes that school should provide more information on prevention of cancer. The young girls need to understand the pathogenesis of cervical cancer better and assess their own risks based on credible sources, so they have better perception of seriousness of the disease.

## Electronic supplementary material

Additional file 1: **Knowledge score and stage of change.** (DOC 30 KB)

## Abbreviations

HPV: Human papillomavirus
HK: Hong Kong
GP: General Practitioner
TV: Television.
