# Supplementary material for: Factors influencing adolescent girls’ decision in initiation for human papillomavirus vaccination: a cross-sectional study in Hong Kong
Source: BMC Public Health. 2014 Sep 8;14:925. doi: 10.1186/1471-2458-14-925 (PMC4176578; doi:10.1186/1471-2458-14-925)
Supplement: Supplementary file 1 — Additional file 1: Knowledge score and stage of change. (DOC 30 KB) [file 12889_2014_7061_MOESM1_ESM.doc]

**Additional file 1** Knowledge score and stage of change

| **Items included in the calculation of knowledge score (total score = 6)** | |
| --- | --- |
| 1. Cervical cancer is a common cancer among women in Hong Kong. 2. Cervical cancer is a kind of diseases can hardly be treated. 3. Having multiple sex partners increases the chance getting cervical cancer. 4. Cervical cancer can develop in any woman who has had sex 5. Cervical cancer can be prevented through Pap’s smear test. 6. Infection by Human Papillomavirus (HPV) is one of the cause for developing cervical cancer. | |
| **Coding of questions according to stage of change** | |
| **Response** | **Stage of change** |
| Have received HPV vaccine | **Action** |
| Considering to be vaccinated | **Intention** |
| Require more information for decision | **Contemplation** |
| Will not consider/has never considered to be vaccinated | **Pre-contemplation** |
